# Simplified and Low-Cost Characterization of Medium-Voltage Low-Power Voltage Transformers in the Power Quality Frequency Range

**DOI:** 10.3390/s22062274

**Published:** 2022-03-15

**Authors:** Alessandro Mingotti, Christian Betti, Lorenzo Peretto, Roberto Tinarelli

**Affiliations:** Department of Electrical, Electronic and Information Engineering, Guglielmo Marconi Alma Mater Studiorum, University of Bologna, Viale del Risorgimento 2, 40136 Bologna, Italy; christian.betti@studio.unibo.it (C.B.); lorenzo.peretto@unibo.it (L.P.); roberto.tinarelli3@unibo.it (R.T.)

**Keywords:** power quality, low-power voltage transformers, characterization, uncertainty, accuracy, low-cost procedure, distribution network, medium voltage

## Abstract

The distribution network is experiencing a massive deployment of intelligent electronic devices (IEDs) such as energy meters, protective devices, and phasor measurement units (PMUs). This phenomenon resulted, on the one hand, in (i) the availability of distributed measurement systems capable of monitoring and collecting measurements from the distribution network, and (ii) increasing awareness of the system operator about the status of the network. On the other hand, such a significant number of devices require to be characterized, over the years, and assessed in both sinusoidal and distorted conditions. However, the characterization process may require a huge investment of money and time considering the low availability of reference instruments and accredited laboratories. To this purpose, this paper presents a simple and fast test procedure, performed with cheap and low-voltage instrumentation, to characterize two off-the-shelf low-power medium-voltage sensors in the power quality frequency range. In detail, the paper describes the measurement setup developed for the characterization and the performed tests. In addition, the method was also reproduced with reference equipment for validation purposes. Lastly, for both tests, an uncertainty evaluation was performed to quantify the goodness of the proposed method. From the results, it is possible to appreciate that the designed cheap and simple test can achieve as accurate results as those of a sophisticated and expensive equipment.

## 1. Introduction

In the last few years, the distribution network (DN) has undergone a huge revolution. System operators (SOs) are day after day more interested in having complete knowledge of their infrastructure due to (i) the availability of real-time data coming from the grid being crucial for its active control during normal and faulty operations, and (ii) the monitoring of specific parameters allowing to run processes of predictive maintenance [1], which contribute to a reduction in the intervals of energy not supplied to the customers. It is than clear that the attention of SOs is becoming more and more DN-oriented compared to the past. As a matter of fact, the transmission network has always been better managed due to its significance in the power system scenario (and, of course, due to higher fund availability).

Therefore, what has been experienced in DNs is a massive deployment of intelligent electronic devices (IEDs) such energy meters, protective devices, phasor measurement units (PMUs), and sensors. The result is the availability of distributed measurement systems (DMSs) capable of assessing the network conditions. For example, PMUs are used, thanks to their synchrophasor measurements, to have an accurate state estimation or voltage control of the network [2,3,4,5]. DMSs are used to run algorithms for the fault location or to assess the health status of the electrical assets (see [6,7] and [8,9,10], respectively). Lastly, energy meters are crucial for triggering protection [11], for running asynchronous algorithms [12], and for their cheapness, which make them suitable for the massive deployment in low-voltage (LV) networks [13].

Despite the numerous benefits brought by the deployment of IEDs and sensors, there are some drawbacks that should be considered and tackled. Firstly, the standards need to be improved to define and regulate the features, testing conditions, and operations of such new assets. Secondly, which is the aim of the paper, the deployed instrumentation needs to be characterized and regularly assessed to confirm its accuracy and reliability. However, the low availability of (i) accredited laboratories to test them, (ii) time and money invested by SOs, and (iii) simple and fast testing procedures hinders asset characterization and, hence, performance.

To this purpose, this paper presents a smart and low-cost characterization procedure for medium-voltage (MV) sensors. LV affordable equipment is used to characterize low-power voltage transformers (LPVTs) in the power quality frequency range (up to 2500 Hz). This work does not focus on the 50 Hz characterization considering that (i) several studies are available on the topic, and (ii) the 50 Hz characterization at rated voltage is one of the main tests to be performed according to the standards. 

The added value of the proposed solution is that the sensors under test can be characterized without applying the rated voltage (typically 20/√3 kV) but with voltage amplitudes below 100 V. Furthermore, an uncertainty evaluation process, performed with the Monte Carlo method (MCM), is provided to support the applicability of the developed testing procedure.

Of course, the characterization of instrument transformers (ITs) is a well-tackled topic in the recent literature. For example, the characterization of inductive VTs was addressed in [14,15,16]. On the other hand, inductive CTs were studied and characterized in [17,18]. Note, that studies on inductive VTs and CTs have been published for a few decades; hence, they can be considered a consolidated topic. As for the LPITs, different kinds of LPCTs and LPVTs were characterized in [17,19,20] and [14], respectively. 

To the authors’ knowledge, the LV solution for the characterization of LPITs was only addressed in [21,22]. In the former study, authors introduced and described a low-cost and versatile acquisition system which can be used for a variety of ITs. In the latter study, the authors instead presented a low-cost procedure for the characterization of inductive VTs based on harmonic compensation. Therefore, it can be concluded that this work contributes to the existing, but poor, literature presenting new and simple ideas to characterize LPVTs in ways that have not previously been tackled.

This work was supported and motivated by the EURAMET project number 19NRM05 entitled “measurement methods and test procedures for assessing accuracy of instrument transformers for power quality measurements” IT4PQ. 

The remainder of this paper is structured as follows: Section 2 contains a summary of the goals and the motivation of the IT4PQ project. Section 3, the core of the work, describes the characterization procedure introduced in this work. First, the measurement setup designed for the test is presented; afterward, the focus is moved toward the tests for validating the entire characterization procedure. Section 4 contains the uncertainty evaluation procedure and its assessment. Lastly, Section 5 is the conclusion of the work.

## 2. IT4PQ Framework

### 2.1. The Consortium

The IT4PQ project is guided by the Italian national metrological institute (NMI) INRIM, and it consists of several other research institutes and NMIs. The involved nations are Italy, Czech Republic, France, Germany, the Netherlands, and Turkey. The overall duration of the project is 36 months. Within this period, the work will be performed and divided into five work packages (WPs) . Each WP will deal with a specific need.

### 2.2. The Project Goals

The need for this project is a consequence of the European direction of decarbonization fixed for 2050. To achieve such goal, the power network should evolve in the renewable direction to become green and efficient. However, this process will arise some issues to be faced in the short term. For example, the presence of disturbances will significantly increase, degrading the so-called power quality (PQ) of the network [23,24]. To this purpose, effective PQ monitoring through accurate measuring instrumentation is needed. This is where IT4PQ starts its work on improving the state of art on PQ monitoring and knowledge.

The project goals can be summarized as follows:

- Definition of accuracy limits for ITs and LPITs in PQ measurements. Such limits will be fixed after their experimental validation.
- Establishment of reference systems and testing conditions capable of characterizing ITs and LPITs in order for them to perform reliable PQ measurements.
- Evaluation of ITs, during PQ measurements, in the presence of multiple influence quantities and factors (temperature, humidity, proximity effects, vibrations, etc.).
- Contribution, with the obtained results, to the development of new versions of the existing standards, e.g., IEC 61869-103 [25], -1 [26], and -6 [27]. The three documents deal with PQ measurements with ITs, general requirements for ITs, and general requirements for LPITs, respectively.

## 3. Simplified and Low-Cost Characterization

This section contains the complete description of the presented characterization process. Firstly, in Section 3.1 the measurement setup designed for the low-cost characterization is introduced. Secondly, in Section 3.2, it is described how the setup components are characterized to be used as a reference. Thirdly, in Section 3.3, the designed low-cost characterization procedure is explained in detail. Lastly, Section 3.4 shows the obtained results that are further manipulated in Section 4 to confirm the efficacy and applicability of the designed procedure.

### 3.1. Measurement Setup

**Figure 1:**
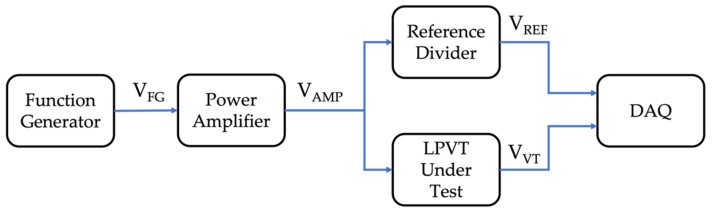
Proposed measurement setup.

The measurement setup designed and adopted for the low-cost and simplified characterization procedure is schematized in Figure 1.

It consists of the following elements:

- Function generator (FG). An arbitrary FG RIGOL DG1022 is used to generate sinusoidal low-voltage signals at different frequencies. Its output voltage is referred to as VFG. The FG can provide sinusoidal signals from 1 µHz to 25 MHz, with an accuracy of ±1 ppm of the setting value and a resolution of 1 µHz.
- Power amplifier. The analog device ADHV4702-1 precision operational amplifier (OA), used with its evaluation board, is used to increase the FG voltage to a more suitable level for the testing (referred to as V_AMP_). The main features of the OA are listed in Table 1.
- LPVT. In this work, two off-the-shelf MV LPVTs are used for testing. One is active and one is passive. Therefore, from hereon after, they are referred to as A and P, respectively. Their main characteristics are summarized in Table 2. As it can be noted from the table, the two devices, despite one being active and one being passive, are quite similar in terms of electrical parameters. They share the same accuracy class (AC), allowing the results of the characterization to be significantly compared and assessed.
- Reference divider. This is a custom-made active resistive divider. Its transformation ratio is 240, and it is used as a reference divider. Its characterization is described in Section 3.2. The choice of an active divider was made to avoid loading effect issues.
- Data acquisition system (DAQ). An NI 9239 DAQ board is used to acquire the voltage signals coming from the reference divider and the LPVTs. The two voltages are referred to as V_REF_ and V_VT_, respectively (in accordance with Figure 1). The DAQ features a 24 bit analog-to-digital converter, an operating voltage range of ±10 V, simultaneous sampling at a maximum of 50 kS/s/ch, and an input impedance of 1 MΩ. The DAQ gain error is ±0.03%, while the offset error is 0.008%. As for the noise, it features 70 µV (rms).

Summarizing the introduced equipment and its role, the voltage of an FG is amplified by an OA to obtain LV, which is significant in absolute terms. Afterward, such a voltage is fed to both the LPVT under test and a reference divider. Their output voltages are then collected through a DAQ for further manipulation. 

### 3.2. Characterization of the Reference Divider

Before describing the proposed characterization procedure (see Section 3.3), it is necessary to introduce the characterization of the active resistive divider. This process is mandatory to guarantee the measurement traceability of the designed measured setup. In other words, the process allows the use of the divider as a reference instrument for the characterization process.

#### 3.2.1. Measurement Setup

**Figure 2:**
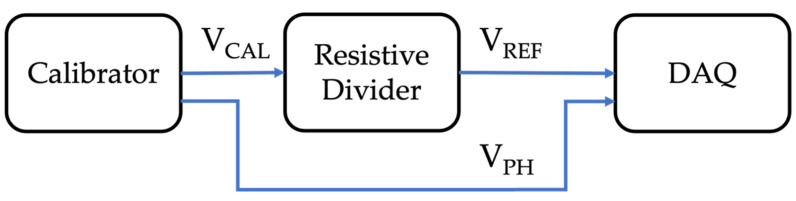
Main features of the fluke calibrator.

The measurement setup used for the characterization is depicted in Figure 2. It consists of a calibrator, a resistive divider, and a DAQ. All components but the calibrator were previously described in Section 3.1. Therefore, its characteristics are listed in Table 3. 

#### 3.2.2. Experimental Characterization of the Divider

The aim is to characterize the reference divider in the frequency range and at the voltage level considered in the characterization procedure described in Section 3.3. The considered frequency range is from 150 Hz to 2000 Hz, and the voltage level is 61.5 V. Details about the reasons of these choices are included in Section 3.3.

With the measurement setup illustrated in Figure 2, 15 sinusoidal signals at different frequencies were applied with the calibrator to the resistive divider. For every frequency, 100 measurements of 10 periods of the single-tone signal were acquired and post-processed. During the characterization process, a second input from the calibrator was acquired to have a reference signal to compute the phase displacement of the resistive divider.

Starting from the acquired voltage V_REF_ and the input of the calibrator V_CAL_, it is possible to obtain the transformation ratio $k_{RD}$ and the phase displacement introduced by the divider $\varphi_{RD}$. Both parameters are extracted from the waveform by means of the Fourier transform.

**Figure 3:**
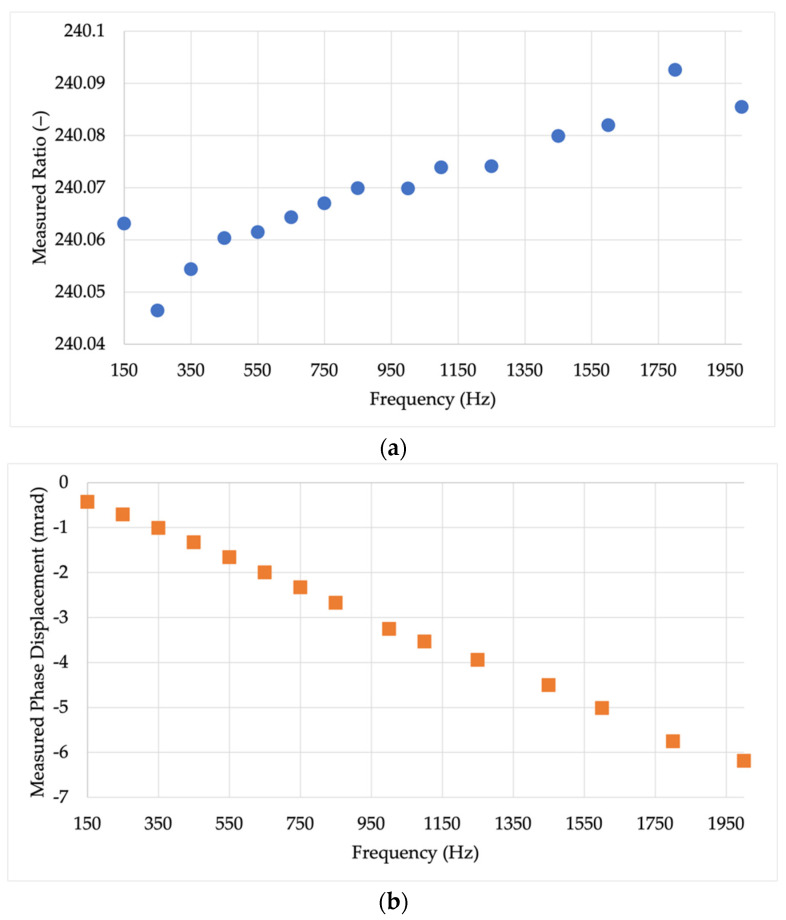
Results of the resistive divider characterization: (**a**) ratio and (**b**) phase displacement.

The results of the resistive divider characterization are presented, in terms of $k_{RD}$ and $\varphi_{RD}$, in Figure 3.

Note that $k_{RD}$ is quite stable over frequency, while $\varphi_{RD}$ increases by a few milliradian as the frequency increases. However, the characterization of the divider allows compensating for its behavior during the main test of Section 3.3.

To quantify the combined uncertainty of the reference divider, it varies from 0.6 *v/v* to almost 1 *v/v* depending on the frequency value. For each case, in Section 4, the adequate combined uncertainty is used for the Monte Carlo trials. 

### 3.3. Test Procedure

#### 3.3.1. Need for Testing

One of the main issues when dealing with IT characterization is the application of high-frequency components at realistic values. As a matter of fact, according to EN-50160 [28], the limits for the harmonic components are, for example, 5.0%, 6.0%, 5.0%, and 3.5%, for the third, fifth, seventh, and 11th harmonics, respectively. Consequently, starting from a 20 kV/√3 rated voltage, the previous values result in harmonic components of around 700 V, 580 V, and 404 V, respectively. Such values are often too high to be reproduced with accurate or simple equipment. This is even more complicated if the harmonic components need to be superimposed with the rated voltage.

To this purpose, the procedure aims at introducing a simple test that allows characterizing the ITs over the power quality frequency range. The designed procedure adopts very low voltages to avoid the use of any complicated or expensive procedure/equipment.

#### 3.3.2. The Procedure

Referring to Figure 1, the idea is to apply an LV to the LPITs under test at the different frequency components. The FG provides an RMS voltage V_FG_ of 3 V, which results in a V_AMP_ of 61.5 V. The reason for this choice is mainly due to the OA. In fact, according to the market, the selected OA is a tradeoff between cheapness and performance. Therefore, the combination FG + OA becomes affordable and suitable for most of the research and manufacturer laboratories. 

At this point, V_AMP_ is applied to both the reference divider (previously characterized) and the LPVT under test. As for the applied voltage, a single tone set of signals was chosen as representative of realistic harmonic components (of a 50 Hz frequency in our case). The selected frequencies were 150 Hz, 250 Hz, 350 Hz, 450 Hz, 750 Hz, 1000 Hz, 1250 Hz, 1600 Hz, and 2000 Hz. Note that the 2000 Hz signal corresponds to the 40th harmonic of the 50 Hz frequencies. Hence, considering that [28] does not provide limits over the 25th harmonics, it can be considered an acceptable top limit.

Turning to the acquisition of the signals, for every signal, 100 measurements of 10 periods were collected from the LPVT under test and the reference divider. Afterward, Fourier transform was applied to extract the rms component and the phase angle for the calculation of the ratio error ε and phase displacement Δφ. The two parameters are defined as

(1) $$\varepsilon =\frac{K_{r}U_{S}-U_{P}}{U_{P}}\times 100,$$

(2) $$\Delta \varphi =\varphi_{S}-\varphi_{P}.\;$$

The two equations feature the rated transformation ratio $K_{r}$, the rms voltages of the primary and secondary signals, $U_{P}$ and $U_{s}$, respectively, and the phase of the primary and secondary signals, $\varphi_{P}$ and $\varphi_{S}$, respectively. Specifying the considered case, the primary signal is measured by the reference divider, while the secondary signal is collected from the device under test.

#### 3.3.3. The Validation Procedure

**Figure 4:**
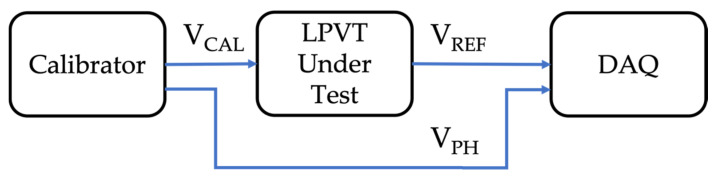
Measurement setup adopted for the reference test.

To understand if the designed procedure is applicable and accurate enough to be adopted, a reference test is needed. Therefore, the measurement setup shown in Figure 4 was used to perform the reference test.

The test has a dual purpose: testing the device with a reference device at the specific voltage and set of frequencies defined in Section 3.3.2, and extending the validation to voltage levels compatible with the standard [28]. In other words, with the calibrator, different voltages are applied (see Table 4), ranging from the low value used in the proposed test (61.5 V) to the max value fixed by [28]. Such a value depends on the harmonic order, and it is listed in Table 4 together with the voltage applied in the reference test. Note, that the percentages taken from [28] refer to the rated voltage, which in our case was 20 kV/√3. 

From the table, it can be observed that the selected value for the designed test (61.5 V) is (i) coherent with the limits of [28] for some harmonic orders, and (ii) quite small compared to the limits of some harmonic orders. Therefore, it consists of a value easier to reproduce and apply.

### 3.4. Experimental Results

All the obtained results are listed in Table 5. Analogously to Table 4, it contains, for each signal (hence, for each frequency), the applied voltage levels. For such voltages, the results are presented, for both LPVTs A and P, in terms of mean values of ε and Δφ. The table includes the results of both the proposed characterization (referred to as “Prop” in the table, in which 61.5 V is always the applied voltage) and the validation test (referred to as “Ref” in the table). However, the notation is adopted to help the reader even though the proposed test was performed only at 61.5 V. 

The values of ε and Δφ are written in Table 5 with a number of significant digits coherent with their associated standard deviation. As it can be seen, this varies from case to case, due to the variation of the frequency and due to the type of the tested LPVT.

At a glance, from the table, it can be qualitatively observed that the values obtained from the designed characterization test are well aligned with those of the reference test.

However, to quantify the goodness of the results, Table 6 and Table 7 are introduced. Table 6 lists ε and Δφ variations between the proposed approach and the reference one. This is done for the 61.5 V test and for the maximum voltage applied for each frequency. The difference between ε values is labeled as Δε, while the difference between Δφ values is labeled as ΔΦ. Note that Δε values are obtained merely as the difference between two percentages; hence, they are still percentages.

Table 7 recalls the accuracy limits fixed by [27] when harmonic measurements are concerned.

Analyzing the limits given in Table 7, it can be concluded that the results in Table 5 are far lower than the limits. In detail, the obtained values are up to four orders of magnitude lower than the limits. This confirms the applicability and validity of the proposed characterization procedure. In fact, the approximation introduced by the proposed approach is significantly lower than the limits defined by the standard for the typical 0.5 accuracy class.

Another comment is on the accuracy level of the obtained results. The simple and low-cost procedure can achieve high standards of accuracy despite the use of common equipment (with low accuracy features). Note also that a full study on uncertainty is provided in Section 4.

## 4. Uncertainty Evaluation

This section aims at quantifying, in a rigorous way, the effectiveness of the proposed characterization procedure by considering all the sources of uncertainty. Such a procedure is known as uncertainty propagation. It is typically implemented by measurists and other researchers to effectively quantify whether a measurement setup is suitable for a specific measurement.

Among the techniques that can be implemented to perform an uncertainty propagation, the selected one is the Monte Carlo Method (MCM). Its details are collected and explained in the guide to the expression of uncertainty in measurements (GUM) [29] and in its Supplement 1 [30].

In the literature, the MCM is widely adopted for a variety of topics, which are not limited to power systems or even engineering. For example, it was applied in [31,32] for line parameter estimation and in [33] for load flow estimation algorithms. The MCM was applied in [34,35] for the evaluation of electrical quantities. Lastly, the application of the MCM allowed to evaluate the accuracy of two setups in [36,37].

In what follows, the MCM is applied to the measurement setup adopted for the proposed characterization procedure and for the reference testing. This, once the sources of uncertainty are known, allows obtaining the confidence intervals associated with the measurement results (ε and Δφ).

### 4.1. Sources of Uncertainty

To identify and understand the sources of uncertainty, Figure 1 and Figure 4 are needed. From Figure 1, it can be noted that there are two contributions to the uncertainty of the proposed measurement setup: the DAQ and the reference divider. To quantify such contributions, the reader can refer to Section 3.1 for the DAQ accuracy parameters and to Section 3.2 for the reference divider characterization results. No other sources of uncertainty exist in the adopted measurement setup.

As for Figure 4, there are again two contributions to the uncertainty inside the measurement setup used for the reference tests: the DAQ and the calibrator. Therefore, the accuracy parameters given in Section 3.2 apply.

### 4.2. MCM Application

Once the sources of uncertainty are known, it is possible to implement the MCM. The procedure consists of corrupting the collected voltages and then computing ε and Δφ. As an example, in the case of the reference measurement setup, the uncertainty propagation is performed for cases where (i) the primary voltage is corrupted by the calibrator accuracy parameters, (ii) the ratio of the LPVT is the rated one, and (iii) the secondary voltage is corrupted by the DAQ accuracy parameters. 

Afterward, 100,000 Monte Carlo trials are performed to compute ε, Δφ, and their 95% confidence intervals. The same process is applied to both the proposed and the reference measurement setups, as well as both LPVTs under test.

The MCM results are then used to assess the coherency and compatibility between the methods, confirming or not the applicability of the proposed one.

### 4.3. MCM Results and Discussion

**Figure 5:**
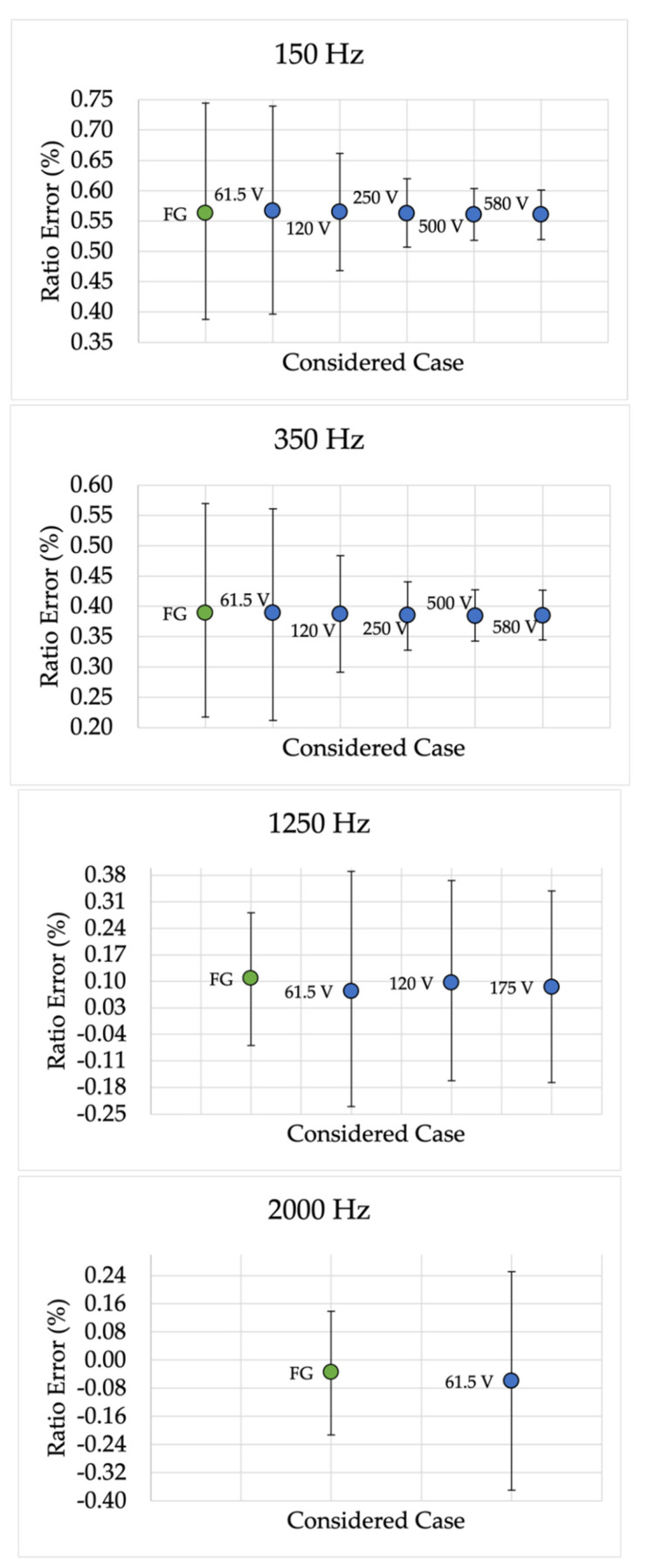
Ratio error 95% confidence intervals for different frequencies and for device A.

**Figure 6:**
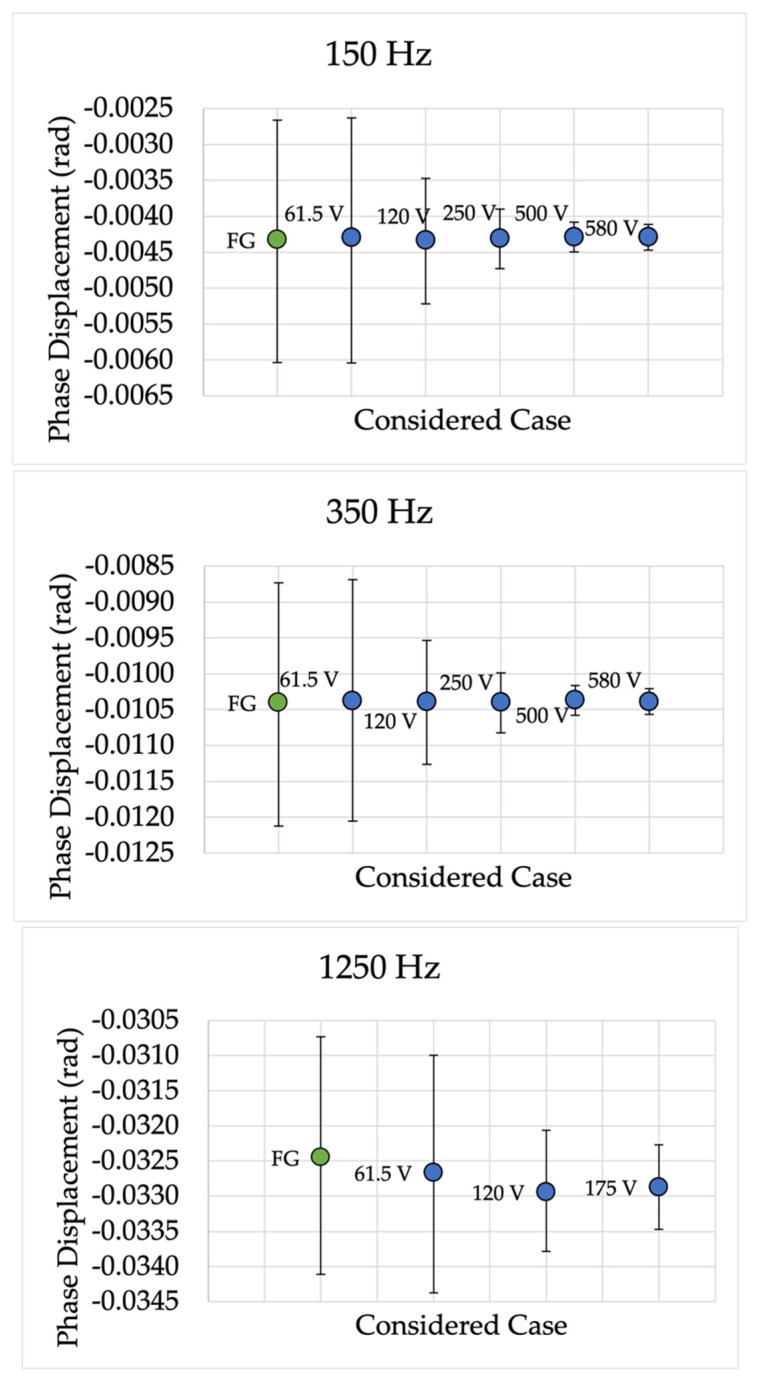

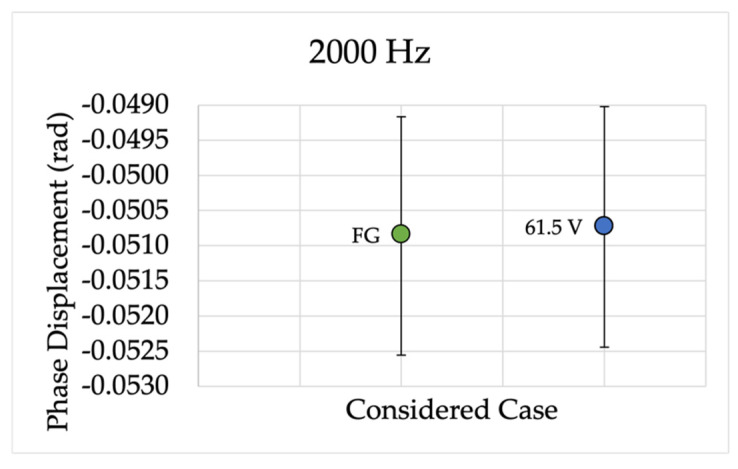
Phase displacement 95% confidence intervals for different frequencies and for device A.

**Figure 7:**
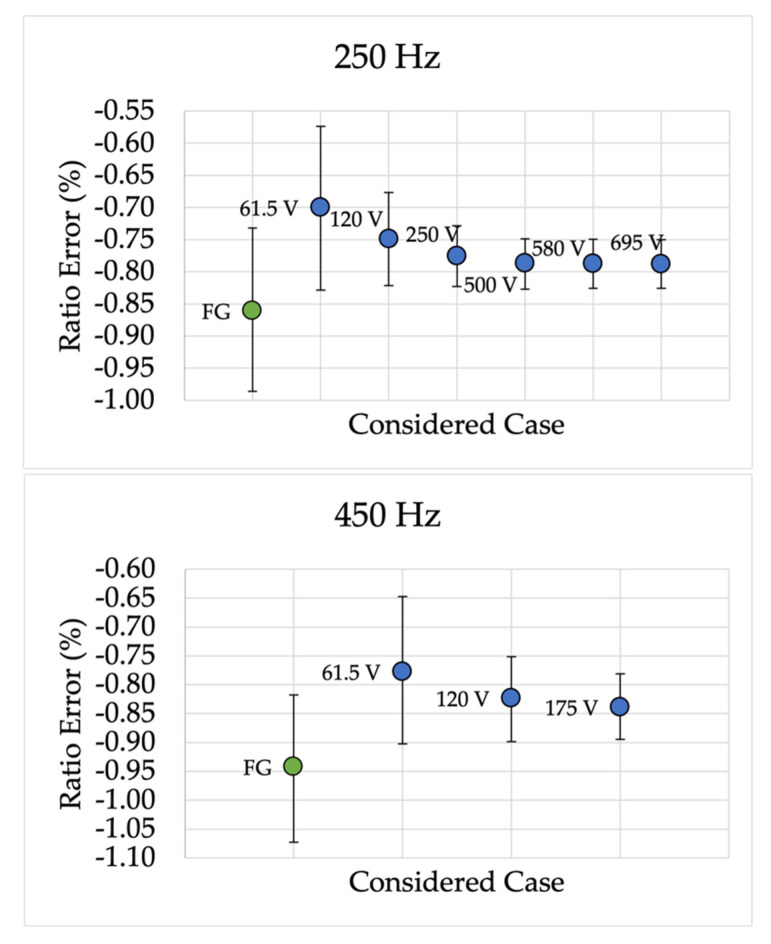

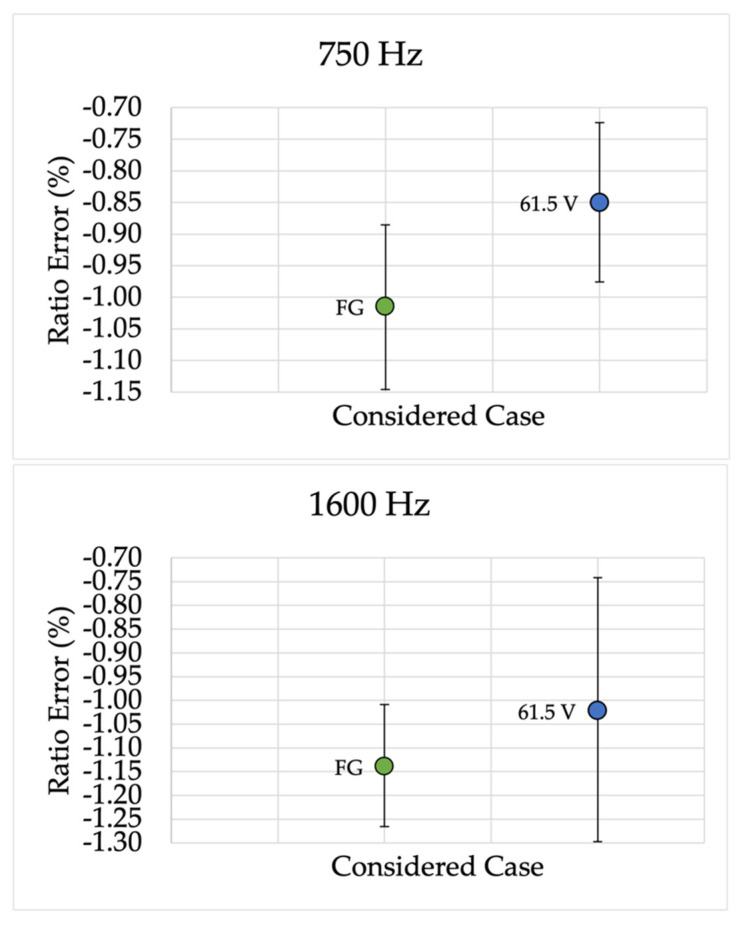
Ratio error 95% confidence intervals for different frequencies and for device P.

**Figure 8:**
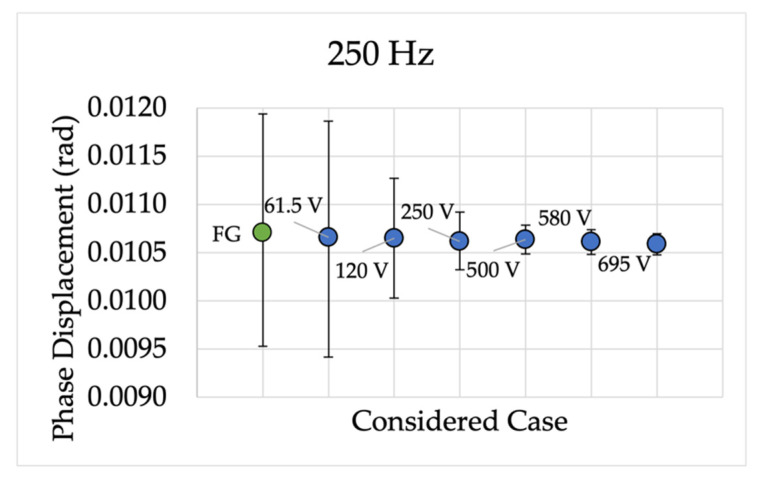

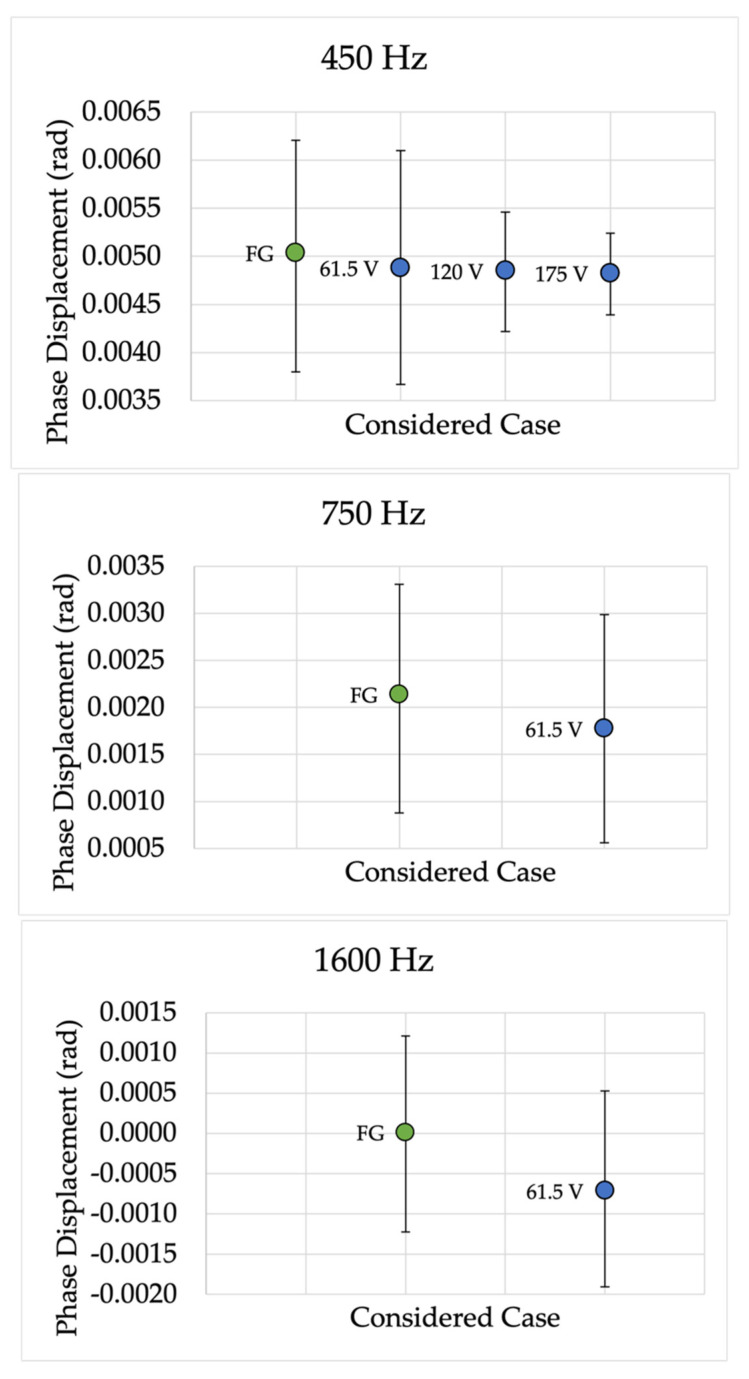
Phase displacement 95% confidence intervals for different frequencies and for device P.

In accordance with the results of the tests listed in Table 5, the graphs below contain the confidence intervals for every performed test. In detail, Figure 5 and Figure 6 show the graphs for ε and Δφ, respectively, in the case of device A. With the same logic, Figure 7 and Figure 8 represent the results for device P.

To increase the readability of the graphs, the following aspects should be considered:

- Each graph illustrates one frequency.
- Blue dots are dedicated to the reference tests (with the calibrator), while green dots are dedicated to the proposed test (function generator plus amplifier).
- Next to each dot, for the sake of clarity, the voltage level of the test is given.
- The confidence intervals, obtained with the MCM, include both type A and type B methods to evaluate uncertainty.
- To keep the length of the results reasonable, not all frequencies are plotted for each device.

Starting from Figure 5, from top to bottom the results for frequencies 150 Hz, 350 Hz, 1250 Hz, and 2000 Hz are plotted. The first comment is on the blue dots. By comparing their intervals, the test performed at the maximum level allowed by the standard is completely coherent with that performed at 61.5 V. This ensures the validity of the selected reference system. A second comment is dedicated to the proposed characterization test. For all frequencies, from 150 Hz to 2000 Hz, the intervals overlap with all other intervals of the reference tests (including for the frequencies not plotted and for all amplitudes). Consequently, it can be concluded that, for the evaluation of ε, the proposed test is effective, accurate, and applicable.

Turning to Figure 6, the Δφ 95% confidence intervals for the same frequencies plotted in Figure 5 are given. From the graphs, it emerges that, in the case of Δφ, all the results are also coherent with those of the reference tests. Furthermore, as introduced in Section 3, the obtained absolute values are far below the limits listed in Table 7. Again, such comments can also be extended to the frequencies not reported in Figure 6.

After the assessment of device A, it can be concluded that the designed characterization procedure is suitable and applicable to active LPVTs. In addition, the method is highly accurate compared to the accuracy limits provided by the standard. 

Analogously to those presented for device A, Figure 7 and Figure 8 collect the device P results for ε and Δφ, respectively. This set of results, for the sake of variety, plots the frequencies not reported in Figure 5 and Figure 6. Hence, the frequencies 250 Hz, 450 Hz, 750 Hz, and 1600 Hz are shown.

From the ε results, it can be observed the coherence with the results obtained for device A. In fact, for all tested frequencies, the proposed characterization procedure is accurate and effective across the range from 150 Hz to 2000 Hz. Note that, as for device A, the absolute values obtained for ε are all far below the limits defined in Table 7.

The last set of comments is dedicated to Δφ, and they refer to Figure 8. As it is observable, the calculated 95% confidence intervals overlap with each other, confirming the effectiveness of the proposed characterization procedure.

In light of all described results, it is possible to conclude that, for both active and passive LPVTs, the proposed characterization procedure is accurate and effective. The goodness of the results is also increased considering the spread of LPVTs inside the power network. In fact, they are becoming the first choice of SOs for measuring and protecting purposes.

Another positive aspect of the proposed characterization procedure is its applicability in the power quality frequency range. Such a range is the main focus of SO, and the instrumentation characterization in that range is extremely important for their correct operation. Furthermore, there are several studies in the literature that tackled the characterization at 50/60 Hz. Hence, the new technique, performed at LV and without expensive or complicate equipment, should facilitate the work of both SOs and researchers.

## 5. Conclusions

The challenge tackled in this work is the characterization of medium-voltage LPVTs. The characterization procedure proposed is performed with low-cost, LV, and common equipment in the power quality frequency range. However, the complete uncertainty evaluation undertaken in the procedure allowed us to confirm its accuracy, effectiveness, and applicability. The evaluation was performed, via Monte Carlo method, on the typical accuracy parameters ratio error and phase displacement. The presented characterization process can simplify than the periodical characterization of LPVTs, thereby increasing the accuracy standard of the equipment installed among the power network. Lastly, the obtained results show how the new simplified procedure can facilitate LPVT characterization. Therefore, this work also provides new inputs to the future versions of the relevant standards.

## Figures and Tables

**Table 1:** Main features of the operational amplifier.

**Dual supply**	±12 V to ±110 V	**CMRR**	160 dB
**Slew rate**	74 V/µs	**Input bias current**	Max 2 pA
**Input noise**	8 nV/√Hz	**Bandwidth**	10 MHz

**Table 2:** Main characteristics of the two LPVTs under test.

Feature	A (Active)	P (Passive)
Primary voltage (kV)	20/√3	20/√3
Rated transformation ratio (-)	20/√3	8200
Rated burden (MΩ)	–	1
Accuracy class	0.5	0.5
Rated frequency (Hz)	50	50
Auxiliary supply (V)	±12	–

**Table 3:** Contains all the relevant features of the Fluke 6105A calibrator.

Range (V)	Frequency (Hz)	Harmonic Distortion		Nonharmonic Noise Floor (%)
		% Setting	% Range	
90	16 to 850	0.016	0.003	0.025
	850 to 6000	0.25	0.015	0.025
180	16 to 850	0.016	0.003	0.016
	850 to 6000	0.25	0.015	0.016
360	16 to 850	0.016	0.003	0.05
	850 to 6000	0.25	0.015	0.05
650	16 to 850	0.016	0.003	0.1
	850 to 6000	0.25	0.015	0.1
1008	16 to 850	0.016	0.003	0.1
	850 to 6000	0.25	0.015	0.1

**Table 4:** Details of the reference tests.

Frequency (Hz)	Harmonic	Limit from [28]	Applied Voltage	
			%	(V)
150	3rd	5.0%	~0.5	61.5
			1.0	120
			2.1	250
			4.3	500
			5.0	580
250	5th	6.0%	~0.5	61.5
			1.0	120
			2.1	250
			4.3	500
			5.0	580
			6.0	695
350	7th	5.0%	~0.5	61.5
			1.0	120
			2.1	250
			4.3	500
			5.0	580
450	9th	1.5%	~0.5	61.5
			1.0	120
			1.5	175
750	15th	0.5%	~0.5	61.5
1000	20th	0.5%	~0.5	61.5
1250	25th	1.5%	~0.5	61.5
			1.0	120
			1.5	175
1600	32nd	0.5%	~0.5	61.5
2000	40th	0.5%	~0.5	61.5

**Table 5:** Measurement results from the characterization procedure and the validation test.

Frequency (Hz)	Applied Voltage		Test	A		P	
	%	(V)		Δ*φ* (Rad)	*ε* (%)	Δ*φ* (Rad)	*ε* (%)
150	~0.5	61.5	Prop	1.9606·10^−2^	−0.8090	−3.89·10^−3^	0.560
			Ref	1.9239·10^−2^	−0.6483	−4.29·10^−3^	0.566
	1.0	120	Ref	1.9218·10^−2^	−0.6928	−4.34·10^−3^	0.565
	2.1	250	Ref	1.91993·10^−2^	−0.71793	−4.308·10^−3^	0.5625
	4.3	500	Ref	1.92005·10^−2^	−0.72828	−4.288·10^−3^	0.5606
	5.0	580	Ref	1.91899·10^−2^	−0.72936	−4.290·10^−3^	0.5603
250	~0.5	61.5	Prop	1.142·10^−2^	−0.869	−6.82·10^−3^	0.43
			Ref	1.066·10^−2^	−0.700	−7.6·10^−3^	0.461
	1.0	120	Ref	1.064·10^−2^	−0.749	−7.65·10^−3^	0.460
	2.1	250	Ref	1.0618·10^−2^	−0.7755	−7.61·10^−3^	0.455
	4.3	500	Ref	1.0635·10^−2^	−0.7866	−7.59·10^−3^	0.453
	5.0	580	Ref	1.0614·10^−2^	−0.7876	−7.61·10^−3^	0.453
	6.0	695	Ref	1.0591·10^−2^	−0.7878	−7.622·10^−3^	0.4542
350	~0.5	61.5	Main	8.063·10^−3^	−0.9120	−9.39·10^−3^	0.382
			Ref	6.96·10^−3^	−0.745	−1.038·10^−2^	0.389
	1.0	120	Ref	6.942·10^−3^	−0.7908	−1.039·10^−2^	0.387
	2.1	250	Ref	6.924·10^−3^	−0.8162	−1.0397·10^−2^	0.3855
	4.3	500	Ref	6.933·10^−3^	−0.8279	−1.0362·10^−2^	0.3843
	5.0	580	Ref	6.902·10^−3^	−0.8289	−1.0388·10^−2^	0.3848
450	~0.5	61.5	Main	6.360·10^−3^	−0.9458	−1.170·10^−2^	0.323
			Ref	4.879·10^−3^	−0.7767	−1.299·10^−2^	0.334
	1.0	120	Ref	4.854·10^−3^	−0.8233	−1.298·10^−2^	0.334
	1.5	175	Ref	4.824·10^−3^	−0.8385	−1.3029·10^−2^	0.3358
750	~0.5	61.5	Main	4.472·10^−3^	−1.0168	−1.813·10^−2^	0.214
			Ref	1.774·10^−3^	−0.8501	−2.062·10^−2^	0.216
1000	~0.5	61.5	Main	4.21·10^−3^	−1.061	−2.34·10^−2^	0.17
			Ref	4.6·10^−4^	−0.952	−2.64·10^−2^	0.13
1250	~0.5	61.5	Main	4.44·10^−3^	−1.091	−2.85·10^−2^	0.11
			Ref	−2.1·10^−4^	−0.987	−3.27·10^−2^	8·10^−2^
	1.0	120	Ref	−1.2·10^−4^	−1.006	−3.29·10^−2^	0.1
	1.5	175	Ref	−2.5·10^−4^	−1.013	−3.287·10^−2^	8.5·10^−2^
1600	~0.5	61.5	Main	5.03·10^−3^	−1.135	−3.618·10^−2^	3.5·10^−2^
			Ref	−7.2·10^−4^	−1.022	−4.126·10^−2^	6·10^−3^
2000	~0.5	61.5	Main	6.07·10^−3^	−1.163	−4.47·10^−2^	−3·10^−2^
			Ref	−1.22·10^−3^	−1.055	−5.07·10^−2^	−6·10^−2^

**Table 6:** ε and Δφ variations between the designed and the reference tests.

Frequency (Hz)	Applied Voltage		A		P	
	%	(V)	ΔΦ (Rad)	Δ*ε* (%)	ΔΦ (Rad)	Δ*ε* (%)
150	~0.5	61.5	3.7·10^−4^	−0.16	4.0·10^−4^	−6.8·10^−3^
	5.0	580	4.2·10^−4^	−8.0·10^−2^	4.0·10^−4^	−6.4·10^−4^
250	~0.5	61.5	7.6·10^−4^	−0.17	7.4·10^−4^	−2.9·10^−2^
	6.0	695	8.3·10^−4^	−8.1·10^−2^	8.0·10^−4^	−2.2·10^−2^
350	~0.5	61.5	1.1·10^−3^	−0.17	9.9·10^−4^	−7.0·10^−3^
	5.0	580	1.2·10^−3^	−8.3·10^−2^	1.0·10^−3^	−3.1·10^−3^
450	~0.5	61.5	1.5·10^−3^	−0.17	1.3·10^−3^	−1.1·10^−2^
	1.5	175	1.5·10^−3^	−0.11	1.3·10^−3^	−1.3·10^−2^
750	~0.5	61.5	2.7·10^−3^	−0.17	2.5·10^−3^	−1.8·10^−3^
1000	~0.5	61.5	3.7·10^−3^	−0.11	3.0·10^−3^	3.5·10^−2^
1250	~0.5	61.5	4.6·10^−3^	−0.10	4.2·10^−3^	3.4·10^−2^
	1.5	175	4.7·10^−3^	−7.8·10^−2^	4.4·10^−3^	2.4·10^−2^
1600	~0.5	61.5	5.7·10^−3^	−0.11	5.1·10^−3^	2.9·10^−2^
2000	~0.5	61.5	7.3·10^−3^	−0.11	6.1·10^−3^	3.3·10^−2^

**Table 7:** Accuracy limits from [27].

**Accuracy Class**	Ratio Error at Harmonics (±%)					Phase Error at Harmonics (±mrad)				
	2nd to 4th	5th and 6th	7th to 9th	10th to 13th	Above 13th	2nd to 4th	5th and 6th	7th to 9th	10th to 13th	Above 13th
0.5	5	10	20	20	+20, −100	87.2	174.5	349.1	349.1	–

## Data Availability

Not applicable.
